# Direct activation of Transient Receptor Potential Vanilloid 1(TRPV1) by Diacylglycerol (DAG)

**DOI:** 10.1186/1744-8069-4-42

**Published:** 2008-10-01

**Authors:** Dong Ho Woo, Sung Jun Jung, Mei Hong Zhu, Chul-Kyu Park, Yong Ho Kim, Seog Bae Oh, C Justin Lee

**Affiliations:** 1 Center for Neural Science, Future Fusion Technology Laboratory, Korea Institute of Science and Technology (KIST), Republic of Korea; 2 Neuroscience Program, University of Science and Technology (UST), Republic of Korea; 3 Department of Physiology and Program in Molecular and Cellular Neuroscience, School of Dentistry, DRI, BK21 Program, Seoul National University, Seoul 110-749, Korea; 4 Department of Physiology, College of Medicine, Kangwon National University, Chunchon 200-710, Korea

## Abstract

The capsaicin receptor, known as transient receptor potential channel vanilloid subtype 1 (TRPV1), is activated by a wide range of noxious stimulants and putative ligands such as capsaicin, heat, pH, anandamide, and phosphorylation by protein kinase C (PKC). However, the identity of endogenous activators for TRPV1 under physiological condition is still debated. Here, we report that diacylglycerol (DAG) directly activates TRPV1 channel in a membrane-delimited manner in rat dorsal root ganglion (DRG) neurons. *1*-oleoyl-2-acetyl-*sn*-glycerol (OAG), a membrane-permeable DAG analog, elicited intracellular Ca^2+ ^transients, cationic currents and cobalt uptake that were blocked by TRPV1-selective antagonists, but not by inhibitors of PKC and DAG lipase in rat DRG neurons or HEK 293 cells heterologously expressing TRPV1. OAG induced responses were about one fifth of capsaicin induced signals, suggesting that OAG displays partial agonism. We also found that endogenously produced DAG can activate rat TRPV1 channels. Mutagenesis of rat TRPV1 revealed that DAG-binding site is at Y511, the same site for capsaicin binding, and PtdIns(4,5)P_2_binding site may not be critical for the activation of rat TRPV1 by DAG in heterologous system. We propose that DAG serves as an endogenous ligand for rat TRPV1, acting as an integrator of G_q/11_-coupled receptors and receptor tyrosine kinases that are linked to phospholipase C.

## Introduction

The capsaicin receptor, TRPV1 (transient receptor potential channel vanilloid subtype 1), is a molecular sensor that detects a wide range of painful stimuli such as capsaicin, heat, and acid in nociceptive sensory neurons [1-4]. Since TRPV1 plays a pivotal role in thermal nociception and inflammatory hyperalgesia [2,3] and is also widely found in the central nervous system [5], considerable effort has been made to identify endogenous activators for TRPV1. The products of lipoxygenases, anandamide, and other endocannabinoids [6-11] and even phosphorylation by protein kinase C (PKC) [12] in the absence of any other agonists have been shown to directly activate TRPV1. However, their roles under physiological condition are still debatable.

Multiple chemical mediators such as bioactive peptides or plasma proteins are generated in inflammatory sites, and many of these mediators heightens the sensitivity of nociceptive sensory neurons after binding to their respective G-protein coupled receptors (GPCR) [13]. Indeed, many Gαq coupled receptors such as bradykinin receptor 2, prostaglandin receptor, protease activated receptor 2, histamine receptor 1, and metabotropic glutamate receptors (mGluR1 and mGluR5), are implicated in sensitization of sensory neurons via TRPV1 modulation during inflammation-induced thermal hyperalgesia [8,14-18]. Diacylglycerol (DAG) is at the core of GPCR signaling pathway and has been shown to directly activate subfamilies of TRP channels. Mammalian homologues of TRP family (TRPC3, C6 and C7) are activated by DAG [19-21], raising the possibility that DAG directly activates TRPV1. Thus, in the present work, we set out to evaluate the possibility of TRPV1 activation by DAG.

## Materials and methods

### Cell preparation and transient transfection

Dorsal root ganglia (DRG) were prepared as previously described [22]. Briefly, Sprague-Dawley rat (OrientBio, Korea) was decapitated, and DRG were rapidly removed under aseptic conditions, placed in HBSS (Gibco). DRG were digested in 0.1% collagenase and 1% collagenase/dispase (Boehringer Mammheim) in HBSS for 10 min respectively, followed by 10 min in 0.25% trypsin (Sigma), all at 37°C. DRG were washed in DMEM (Gibco) 3 times and resuspended in F 12 with 10% FBS (Gibco) and 1% penicillin/streptomycin (Sigma). DRG were then mechanically dissociated with fire-polished glass pipettes, centrifuged, resuspended in F12 media, and then plated on polyornithine (Sigma) and laminin (Sigma)-coated glass coverslips. The cells were maintained at 37°C in 5% CO2 incubator. Human embryonic kidney (HEK) 293 cells (American Type Culture Collection, Manassas, VA) were maintained according to the supplier's recommendations. For transient transfection, cells were seeded in 12-well plates. The next day, 0.5–2 μg/well of pcDNA constructs of TRPV1 or mutants of TRPV1 were transfected into cells using lipofectamine 2000 transfection reagent (Invitrogen) according to the manufacturer's protocol. After 18–24 h, cells were trypsinized and used for whole cell recordings and Calcium imaging experiments.

### Electrophysiology

Whole cell currents were recorded using an Axopatch 200A amplifier (Axon Instruments). Patch pipettes were made from borosilicate glass and had resistances of 3–5 MΩ when filled with standard intracellular solutions. For whole cell experiments, we used an external bath medium (normal Tyrode solution) of the following composition (in mM): 140 NaCl, 5 KCl, 2 CaCl_2_, 1 MgCl_2_, 10 glucose, and 10 *N*-[2-hydroxyethyl]piperazine-*N'*-[2-ethanesulfonic acid] (HEPES), with pH adjusted to 7.4 using NaOH. Cs^+^-rich external solution was made by replacing NaCl and KCl with equimolar CsCl. CaCl_2 _was simply omitted from the external bath medium to produce Ca^2+^-free PSS. The pipette solution contained (in mM) 140 CsCl, 10 HEPES, 5 EGTA, and 3 MgATP, with pH adjusted to 7.3 using CsOH. All drug solutions were applied to cells by local perfusion through a capillary tube (1.1 mm inner diameter) positioned near the cell of interest. The solution flow was driven by gravity (flow rate, ~1–5 ml/min) and controlled by miniature solenoid valves (The Lee Company, Westbrook, CT). The chamber volume was 400 μl, and the time required to reach the chamber was ~30 s. Latency was the time from arrival time of solution in the chamber to the peak activation of current. Currents were filtered at 5 kHz (-3 dB, 4-pole Bessel), digitized using a Digidata 1322 Interface (Axon Instruments), and analyzed using a personal computer equipped with pClamp 9.0 software (Axon Instruments). The calculated junction potential between the pipette and bath solutions used for all cells during sealing was 4 mV (pipette negative, using pClamp 9.0 software). No junction potential correction was applied. Experiments were performed at room temperature (18–22°C).

### Ca^2+ ^imaging

Ca^2+ ^imaging experiment was performed as previously described using fura-2AM (Molecular Probes, Eugene, OR, USA) as the fluorescent Ca^2+ ^indicator. Briefly, cells prepared loaded with fura-2 AM (5 μg) mixed with 5 μl of pluronic acid for 40 min at 37°C in a balanced salt solution [BSS; containing (in mM): 145 NaCl, 5 KCl, 2 CaCl_2_, 1 MgCl_2_, 10 HEPES, and 10 glucose] were plated onto poly-D-lysine-coated coverslips which were mounted onto the chamber (total volume of 500 μl), then placed onto the inverted microscope (Olympus IX70, Japan), and perfused continuously by BSS at a rate of 2 ml/min. All measurements were made at 36°C as controlled by 2-channel temperature controller PTC-20 (ALA Scientific Instrument Inc., USA). Cells were illuminated with a 175W xenon arc lamp, and excitation wavelengths (340/380 nm) were selected by a Lambda DG-4 monochromatic wavelength changer (Sutter Instrument, Novato, CA, USA). Intracellular free Ca^2+ ^concentration ([Ca^2+^]_i_) was measured by digital video microfluorometry with an intensified CCD camera (Cascade, Roper Scientific, Trenton, NJ, USA) coupled to a microscope and a Pentium 5 computer with software (Metamorphor, Universal Imaging Corp., PA, USA).

### Cobalt uptake measurement

Our cobalt uptake staining was modified from Davis [23]. HEK 293 cells were transfected with TRPV1 and GFP and then cultured on glass coverslips. Cells were washed 3 times with uptake buffer (in mM: Sucrose 232, NaCl 5.8, MgCl_2 _2, CaCl_2 _0.25, Glucose 12, Hepes 10). The cells were pretreated for 5 min with the following blockers, 100 μM 6-Iodo *nor *dihydrocapsaicin (6-cap), 2 μM chelerythrine, 20 μM RHC 80267, and 20 μM capsazepine. Cells were then treated for 15 min with solution containing 5 mM cobalt and various drugs. Then the cells were washed with uptake buffer 6 times. The loaded cobalt ions were precipitated with 0.12% Ammonium Sulfide for 5 min and washed with uptake buffer 3 times. The cells were immediately fixed with 4% paraformaldehyde in PBS for 10 min and then washed 3 times in PBS. To develop the precipitated cobalt, cells were incubated with 2% sodium tungstate in uptake buffer for 10 min. During this time, 0.25% ascorbic acid was prepared freshly and mixed with silver nitrate solution. The mixture solution was exposed to the cells to develop the cobalt precipitate as a dark staining. Cells were finally washed and mounted for photography.

### Whole tissue reverse transcription-polymerase chain reaction (RT-PCR)

Total RNA was prepared from HEK 293 cells and DRG neurons with the use of Trizol reagent (Invitrogen, Carlsbad, CA, USA), according to the manufacturer's instructions. cDNA was synthesized with the SuperscriptTM Preamplification System (Invitrogen), as previously described [22]. PCR reaction was performed with 2 μl of the resulting cDNA, with the use of Taq DNA polymerase (Invitrogen), and primers for PCR were 'outer' primer.

### Single-cell reverse transcription-polymerase chain reaction (RT-PCR)

We adopted methods described by Silbert *et al*. [24]. Briefly, following Ca^2+ ^imaging experiments, we harvested cells using patch pipettes (tip diameter, 30 μm) filled with autoclaved distilled water under visual control and then the cell was gently put into a reaction tube containing reverse transcription agents. Optionally, to avoid genomic DNA contaminations, a DNase I (for 40 min at 37°C) digest was performed before reverse transcription. After heat inactivation, RT was carried out for 50 min at 50°C (superscript III, Invitrogen RT). Subsequently, the cDNA was divided into four or five 2 μl aliquots that were used in separate PCRs. All PCR amplifications were performed with nested primers. The first round of PCR was preformed in 50 μl of PCR buffer containing 0.2 mM dNTPs, 0.2 μM 'outer' primers, 5 μl of RT product, and 0.2 μl of platinum Taq DNA polymerase (Invitrogen). The protocol included 5 min of initial denaturation at 95°C, followed by 35 cycles of 40 s of denaturation at 95°C, 40 s of annealing at 55°C, 40 s of elongation at 72°C, and was completed with 7 min of final elongation. For the second round of amplification, the reaction buffer (20 μl) contained 0.2 mM dNTPs, 0.2 μM 'inner' primers, 5 μl of the products from the first round, and 0.1 μl of platinum Taq DNA polymerase. The reaction was the same as the first round. The PCR products were then analyzed on an ethidium bromide-stained 2% agarose gel, and photographed using a digital camera.

### Site-directed Mutagenesis

Point mutant Y511A of TRPV1 was produced with a two step PCR approach from TRPV1 construct which was generated in our lab. In brief, it was generated by a combination of two overlapping PCR fragments, which were constructed from complimentary mutagenic primers. The following primers contained the single point mutation shown in bold (5'-GAGGAGTC**GC**CTTCTTCTTCC and 5'-GGAAGAAGAAG**GC**GACTCCTC). TRPV1 mutant of Δ747–838 was produced by one-step PCR-based method. Briefly, the PCR product containing SacII/KpnI sites was obtained with an internal forward primer (CGCTTACAGCAGCAGTGAGACCC) and a KpnI site-stop codon-tailing reverse primer (TATGGTACCTTACAGGGTGCGCTTGACGCCCTC). The pcDNA3.1 (+)/TPRV1 construct digested with SacII/KpnI was ligated with the PCR product. After mutagenesis, the sequences of the final constructs were confirmed by DNA sequencing.

### Statistics

Results are expressed as mean ± SE. Where appropriate, results were compared using 2-tailed Student's *t*-test (paired or non-paired) or ANOVA test.

## Results

### OAG induces Ca^2+ ^influx in DRG neurons

**Figure 1:**
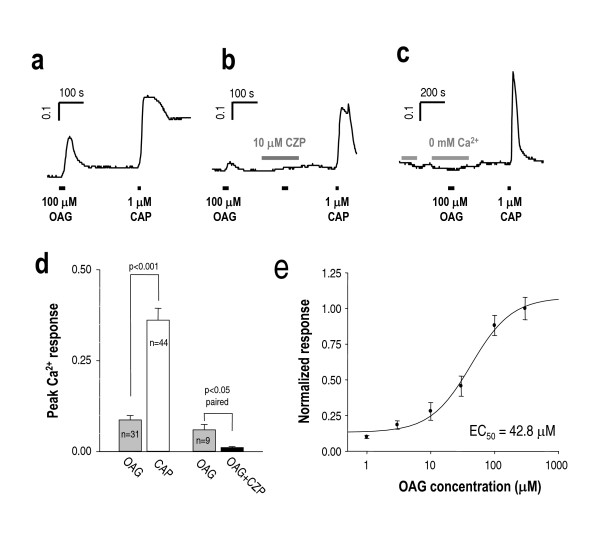
**OAG induces Ca^2+ ^influx in rat DRG neurons that is blocked by capsazepine (CZP). **(a) The representative trace of fura-2 ratiometric Ca^2+ ^imaging reveal activation of TRPV1 by 100 μM OAG in capsaicin (CAP) responsive DRG neurons. Ca^2+ ^transients were evoked by the application of 100 μM OAG, (b) whereas Ca^2+ ^transients were blocked by the pretreatment of 10 μM CZP and (c) of 0 mM Ca^2+ ^in the extracellular solution. The DRG neurons were exposed to 1 μM capsaicin to determine capsaicin-sensitivity in the end of each experiment. (d) Summary of Ca^2+ ^transient responses to 1 μM capsaicin, 100 μM OAG, 100 μM OAG with 10 μM CZP, as measured by peak amplitude of ratio for each transient. The mean value of OAG-induced Ca^2+ ^transient was 0.086 ± 0.012, while that of capsaicin-induced Ca^2+ ^transient was 0.36 ± 0.032. The OAG-induced Ca^2+ ^transient was blocked by 10 μM CZP (0.001 ± 0.003). Each p value was indicated on each bar group. (e) The concentration-response curve of OAG response in DRG neurons obtained from ratiometric Ca^2+ ^imaging.

We first examined whether *1*-oleoyl-2-acetyl-*sn*-glycerol (OAG), a DAG analog, can induce Ca^2+ ^responses in rat DRG neurons. We performed fura-2 ratiometric Ca^2+ ^imaging experiments to verify TRPV1 activation by 100 μM OAG. An increase in [Ca^2+^]_i _was elicited by 100 μM OAG in capsaicin-responsive DRG neurons (Figure 1a), which was abolished by a pretreatment with the TRPV1 blocker, 10 μM capsazepine (Figure 1b) and with 0 mM Ca^2+ ^in the bath solution (Figure 1c), indicating that Ca^2+ ^transients induced by 100 μM OAG is mostly due to the influx of Ca^2+ ^through TRPV1 rather than the mobilization from intracellular Ca^2+ ^stores. Of the 44 DRG neurons that showed a response to capsaicin we found that 31 neurons responded. 13 out of 44 neurons only showed capsaicin response. Less than 5% of the DRG neurons responded only to OAG and these cells possibly represent an OAG activation of other TRPC channels. The summary of peak amplitude showed that OAG-induced Ca^2+ ^transients were about 23% of capsaicin-induced Ca^2+ ^transients, and almost were completely inhibited by capsazepine (Figure 1d). Additionally, concentration-response curve of OAG-induced Ca^2+ ^transient showed that the half-maximal concentration (EC50) of OAG is 42.8 μM (Figure 1e).

### OAG-induced Ca^2+ ^transients correlate RNA expression of TRPV1 in each DRG neuron

**Figure 2:**
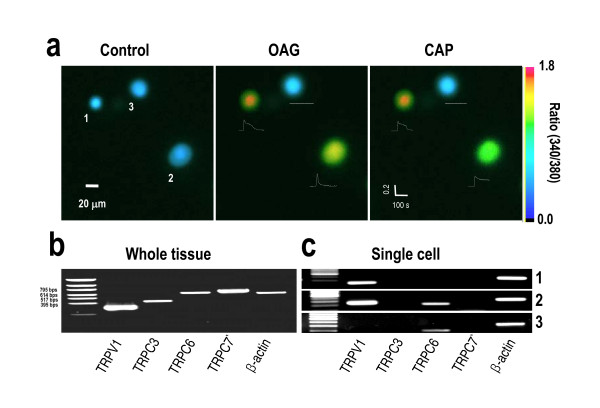
**OAG-induced Ca^2+ ^transients correlate with mRNA expression of TRPV1 in each DRG neuron.** Combination of single-cell RT-PCR following Ca^2+ ^imaging (n = 10) revealed an association between expression of TRPV1 and the responsiveness to both OAG and capsaicin. DRG neurons responsive to 100 μM OAG and 1 μM capsaicin (Number 1 and 2 on (a), n = 6/10) expressed TRPV1 (n = 6/6), whereas DRG neurons unresponsive to 100 μM OAG and 1 μM capsaicin did not (number 3 on a, n = 4/4). Most cells (n = 9/10) expressed TRPC6 and some of them did not respond to 100 μM OAG (n = 4/9). (a) Three images represent fura-2 ratio images before and during 100 μM OAG and 1 μM capsaicin applications. Traces show Ca^2+ ^transients in response to OAG or capsaicin application. (b) Acutely prepared DRG tissues showed the RNA expression level on TRP subfamilies. (c) Single cell RT-PCR results showed the mRNA level of TRP subfamilies. Numbers indicate each cell shown in single cell RT-PCR results.

To determine whether OAG-induced Ca^2+ ^transients are mediated by TRPV1 or by other TRPC members in the TRP family, we performed RT-PCR on whole DRG tissue and individual DRG neurons harvested after testing with 100 μM OAG and 1 μM capsaicin. The results of RT-PCR on the whole DRG tissue showed that the various subtypes of TRP family which are Ca^2+^-permeable channels were detected (Figure 2b). In order to determine the correlation between Ca^2+ ^influx and subtypes of TRP family, single-cell RT-PCR technique was employed. All of DRG neurons responsive to both 100 μM OAG and 1 μM capsaicin expressed TRPV1 mRNA (Figure 2c, n = 6/6), whereas all of DRG neurons unresponsive to both 100 μM OAG and 1 μM capsaicin did not (Figure 2c, n = 4/4). Most cells (Figure 2c, n = 9/10) expressed TRPC6 and some of them responded to 100 μM OAG (Figure 2c, n = 4/9). These results indicated that there was a strong correlation between TRPV1 mRNA expression and OAG-responsiveness. In addition none of these cells expressed TRPC3 or TRPC7 (Figure 2c), even though both TRPC3 and TRPC7 were detected in whole DRG tissue (Figure 2b). There was a lack of correlation between an OAG-induced Ca^2+ ^transient and TRPC6 expression in DRG neurons, since only 4 out of 9 cells expressing TRPC6 showed OAG-induced Ca^2+ ^transient. Taken together, these results demonstrated that DAG-mediated Ca^2+ ^response in DRG neurons is strongly correlated with TRPV1 expression.

### OAG induces Ca^2+ ^transient and inward currents via TRPV1 in HEK 293

**Figure 3:**
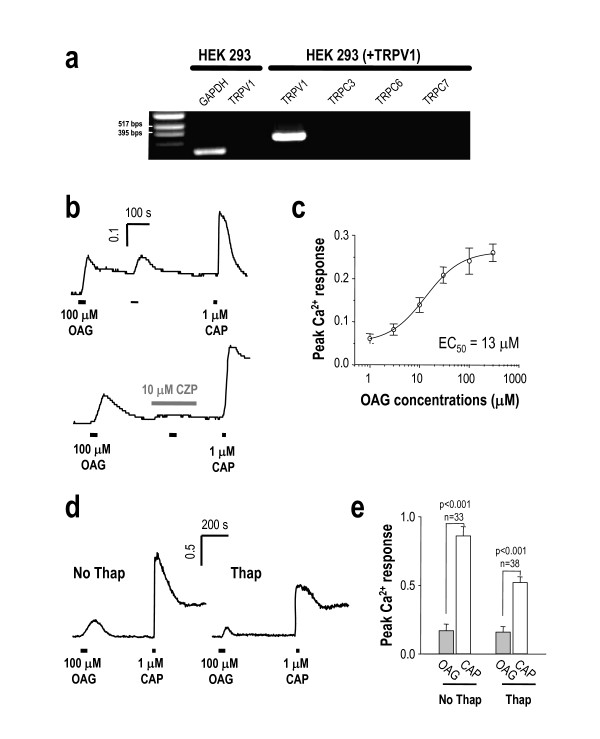
**OAG-induced Ca^2+ ^increase is inhibited by TRPV1 blockers in heterologous expression system.** (a) RT-PCR analysis showed that in untransfected HEK 293 cells, there was no endogenous expression of TRPV1, TRPC3, TRPC6 or TRPC7. TRPV1 expression was detected in TRPV1-transfected HEK 293 cells. Glyceraldehyde 3-phosphate dehydrogenase (GAPDH) was positive control on RT PCR. (a) Ratiometric Ca^2+ ^imaging loaded with fura-2AM revealed activation of TRPV1 by 100 μM OAG. (b) OAG application evoked Ca^2+ ^transients that were blocked by the pretreatment with 10 μM CZP. Capsaicin-induced Ca^2+ ^transients confirmed expression of functional TRPV1 in the transfected HEK 293 cells. (c) Concentration-response relationship of OAG response in TRPV1-expressing HEK 293 cells obtained from ratiometric Ca^2+ ^imaging. (d, e) The OAG-induced Ca^2+ ^transients persist in the presence of 1 μM Thapsigargin (Thap). (f) The summary bar graph was generated from the peak Ca^2+ ^responses of OAG-induced and capsaicin-induced Ca^2+ ^transients in the absence and presence of 1 μM thapsigargin. Although the capsaicin-induced Ca^2+ ^transient was slightly decreased, there was no difference in OAG-induced-Ca^2+ ^transients between both conditions.

To exclude the involvement of TRPC subfamily in OAG-induced responses, we used HEK 293 cells, which do not express TRPC3, TRPC6, and TRPC7 (Figure 3a). In agreement with the Ca^2+ ^responses observed in capsaicin-responsive DRG neurons, Ca^2+ ^imaging data performed in HEK 293 cells showed that an increase in [Ca^2+^]_i _was also induced by treatment of 100 μM OAG in TRPV1-expressing, capsaicin-sensitive cells (Figure 3b, upper panel), and these responses were blocked by treatment with 10 μM capsazepine (Figure 3b, lower panel). In addition, concentration-response curve of OAG-induced Ca^2+ ^transient in TRPV1-expressing HEK 293 cells showed that EC50 is 13 μM (Figure 3c). To test whether the intracellular store of Ca^2+ ^contributes to OAG-induced Ca^2+ ^transients in TRPV1-expressing HEK 293 cells, we blocked Ca^2+ ^release from internal store by treating with 1 μM thapsigargin. We found that 1 μM thapsigargin treatment minimally affected OAG-induced Ca^2+ ^transient. In the presence of 1 μM thapsigargin, the peak ratio values of OAG and CAP-induced Ca^2+ ^transients were 0.15 ± 0.040 and 0.52 ± 0.042 (Figure 3d, e) respectively, while the peak ratio values of those in the absence of thapsigargin were 0.17 ± 0.047 and 0.86 ± 0.067 (Figure 3d, e). The percentage of OAG response to capsaicin response was about 20%, which was similar to that shown in DRG neuron (Figure 1d). Although the average OAG response did not change in the presence and absence of 1 μM thapsigargin, there was a reduction of capsaicin-induced Ca^2+ ^transients in the presence of thapsigargin (Figure 3e). This is probably due the fact that capsaicin causes an increase in [Ca^2+^]_i _not only by Ca^2+ ^entry through TRPV1 but also by releasing Ca^2+ ^from intracellular stores [25].

**Figure 4:**
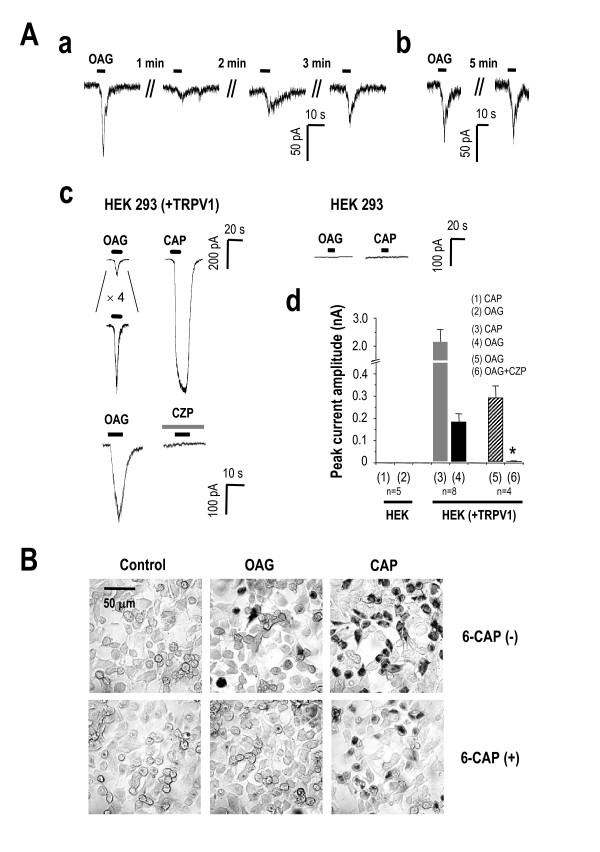
**OAG-induced inward current is inhibited by TRPV1 blockers in heterologous expression system.** (Aa) Currents generated by repetitive 100 μM OAG application showed recovery from desensitization. The intervals of each trace were 1 min, 2 min, and 3 min as indicated. (Ab) The repeated application of OAG currents showed full recovery after 5 min on a different cell. (c, upper panel) 100 μM OAG induced inwarnd current in HEK cell expressing TRPV1 which responded to 1 μM capsaicin. (c, lower panel) 100 μM OAG elicited inward currents that were abolished by 10 μM CZP in TRPV1-transfected cells. OAG did not induce any current in untransfected cells. (d) Summary of the current responses to 1 μM capsaicin, 100 μM OAG, and 100 μM OAG with 10 μM CZP, as measured by peak current amplitude. The asterisk showed significant difference in OAG-induced Ca^2+^transient blocked by CZP (t-test, P < 0.05). (g) Co^2+ ^uptake experiments further verified TRPV1 activation by 100 μM OAG. Co^2+ ^uptake induced by 100 μM OAG (upper middle panel) was abolished by the TRPV1 antagonist, 100 μM 6-iodonordihydrocapsaicin (6-CAP) (lower middle panel). Lower panel shows the same experimental condition for capsaicin.

To test whether OAG is enough to activate TRPV1, we performed whole-cell patch clamp in HEK 293 cells heterogously expressing TRPV1. We found that treatment of OAG induced inward current in TRPV1-expressing HEK 293 cell, which strongly desensitized within a minute and recovered after 5 min (Figure 4Aa). OAG-induced inward currents were observed only in TRPV1-expressing HEK 293 cells but not in untransfected cells, and this current was blocked by 10 μM capsazepine (Figure 4Ac). This summary bar graph showed that OAG-induced inward current was about 20% in capsaicin-responsive cells (Figure 4Ad) and this result suggests that OAG might act as a partial agonist. Other analogs of DAG including 100 μM SAG and 100 μM DOG also evoked inward currents (data not shown), suggesting that these effects are general phenomena associated with DAG analogs.

Next, we performed Co^2+ ^uptake assay using TRPV1-expressing HEK 293 cell since this technique has been extensively used as a functional assay for TRPV1 channel activity [23]. We treated 100 μM OAG and measured Co^2+ ^uptake through TRPV1 expressed in HEK 293 cells. We found that, like capsaicin, 100 μM OAG induced Co^2+ ^uptake although with lower intensity and less number of cells (Figure 4B). The Co^2+ ^uptake by OAG and capsaicin was blocked by TRPV1-selective antagonists, including 50 μM 6-iodonordihydrocapsaicin or 10 μM capsazepine (Figure 4B). Taken together, our results support the idea that OAG could activate TRPV1 and OAG-induced responses in DRG neurons are mediated by TRPV1.

### OAG-induced TRPV1 activation is PKC- and DAG-lipase independent

**Figure 5:**
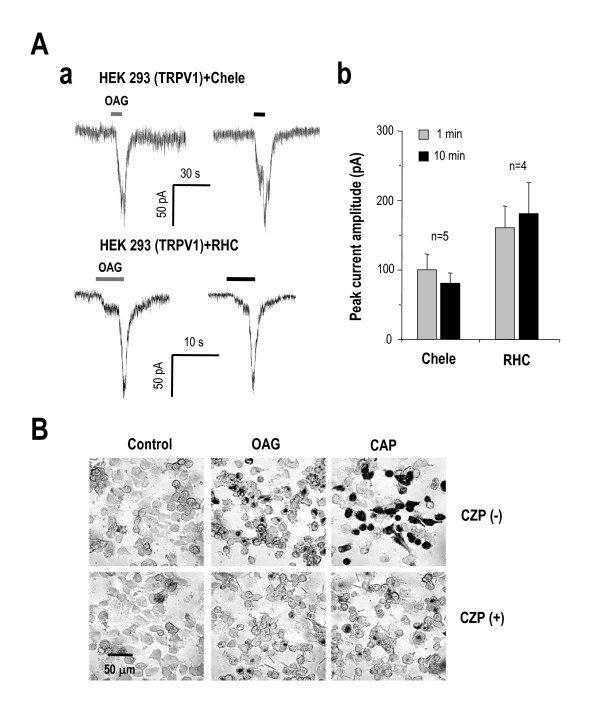
**OAG-induced TRPV1 activation is PKC and DAG-lipase independent.** (Aa) OAG-induced current was well generated in 1 μM chelerythrine, a PKC inhibitor in the internal pipette solution in the whole cell patch mode on the condition of 1 min (left trace) and 10 min (right trace) after rupturing cell. (Ab) OAG-induced current was also examined in 10 μM RHC80267, a DAG-lipase inhibitor in the same manner. (c) The summary bar graph was derived from the current peak amplitude in both conditions a PKC inhibitor and a DAG-lipase inhibitor at each 1 min and 10 min recording data. Each inhibitor was included in the patch pipette solution. (d) Co^2+ ^uptake by OAG in TRPV1 expressing HEK 293 cells were observed even in the presence of 1 μM chelerythrine and 10 μM RHC80267 and was blocked by 10 μM CZP, similar to 1 μM capsaicin.

We next tried to determine the underlying mechanism of OAG-induced TRPV1 activation. Since it has been known that DAG analogs directly activate other members of TRP channels, such as *Drosophila *TRP channels [20,21] and mammalian TRPC channels [19,20], we tested whether DAG analogs can induce TRPV1-meidated currents directly via a membrane-delimited pathway, independent of PKC or DAG-lipase. To eliminate the possible contribution of PKC phosphorylation and the activity of DAG lipase to the OAG-induced responses, we performed whole-cell recordings from HEK 293 cells transiently expressing TRPV1 with a pipette solution containing either 1 μM chelerythrine, a well known PKC inhibitor, or 10 μM RHC80267, a highly selective DAG lipase inhibitor [26]. We found that the application of 100 μM OAG induced TRPV1 current in the presence of 1 μM chelerythrine or 10 μM RHC80267 in the pipette solution, indicating that OAG could induce TRPV1 current in PKC or DAG-lipase independent manner (Figure 5Aa). In order to allow complete diffusion of these inhibitors, OAG was applied twice, at 1 min and 10 min after rupture of the cell membrane. The amplitude of OAG-induced current was similar when OAG applied 1 min or 10 min (Figure 5Aa, b), indicating that PKC or DAG-lipase did not have much role in induction of TRPV1-meidated current by OAG. We independently tested the effective block of PKC by 1 μM chelerythrine in a separate experiment. At this concentration, chelerythrine effectively blocked the tachyphylaxis of TRPV1 upon repetitive stimulation [see Additional file 1], which is known to be caused by PKC phosphorylation of TRPV1 [27]. The Co^2+ ^uptake induced by 100 μM OAG in TRPV1-expressing HEK 293 cells was also discernable even in the presence of 1 μM chelerythrine and 10 μM RHC80267 (Figure 5B), further supporting the direct activation of TRPV1 by 100 μM OAG.

### Endogenously produced DAG activates TRPV1 channels in a heterologous expression system

**Figure 6:**
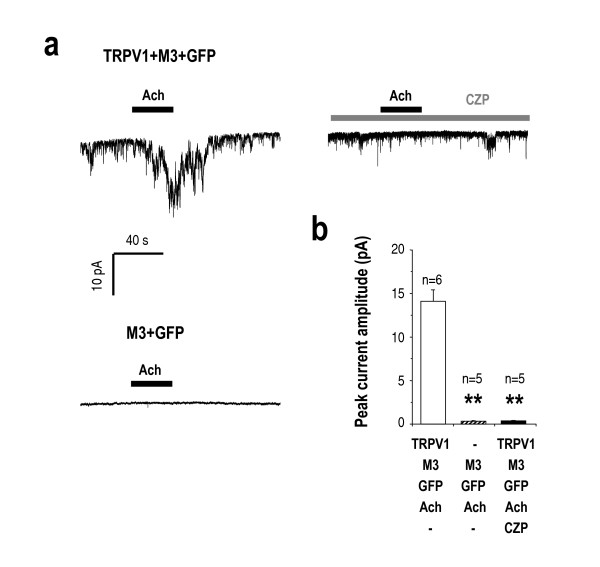
**Endogenously produced DAG activates TRPV1 channels in a heterologous expression system.** (a) Activation of muscarinic receptor M3 by 30 μM Acetylcholine (Ach) opened TRPV1 channels (a, upper left panel) in HEK 293 cells coexpressing M3- and TRPV1 (upper left panel). Patch pipette solution contained both 1 μM chelerythrine and 10 μM RHC80267 Ach-induced currents were almost completely blocked by 10 μM CZP (upper right panel). Ach-induced currents were not observed in HEK 293 cells expressing only M3 (lower left panel). (b) Summary of Ach-evoked currents. Asterisks represent a significant difference by ANOVA test followed by Tukey – Kramer multiple comparison test (n. s: non-significant, ** p < 0.001).

DAG is produced upon activation of G_αq/11_-coupled receptors or a class of receptor tyrosine kinases, which then activates phospholipase C, resulting in the conversion of PtdIns(4,5)P_2 _to DAG and IP_3 _[19]. Thus, we examined whether endogenously produced DAG can activate TRPV1 channels. In HEK 293 cells over-expressing M3, a G_αq/11_-coupled muscarinic acetylcholine receptor, the addition of 30 μM acetylcholine did not induce any significant current (Figure 6a lower left panel). However, in HEK 293 cells expressing both M3 and TRPV1, acetylcholine induced significant inward currents even in the presence of both 1 μM chelerythrine and 10 μM RHC80267 in the pipette solution (Figure 6a upper left panel). The acetylcholine-induced currents were almost completely blocked by 10 μM capsazepine (Figure 6a upper right, b), indicating that the acetylcholine-induced current is mediated by TRPV1. These results provide strong support for DAG acting as an endogenous ligand for TRPV1.

### OAG binds to the capsaicin binding site

**Figure 7:**
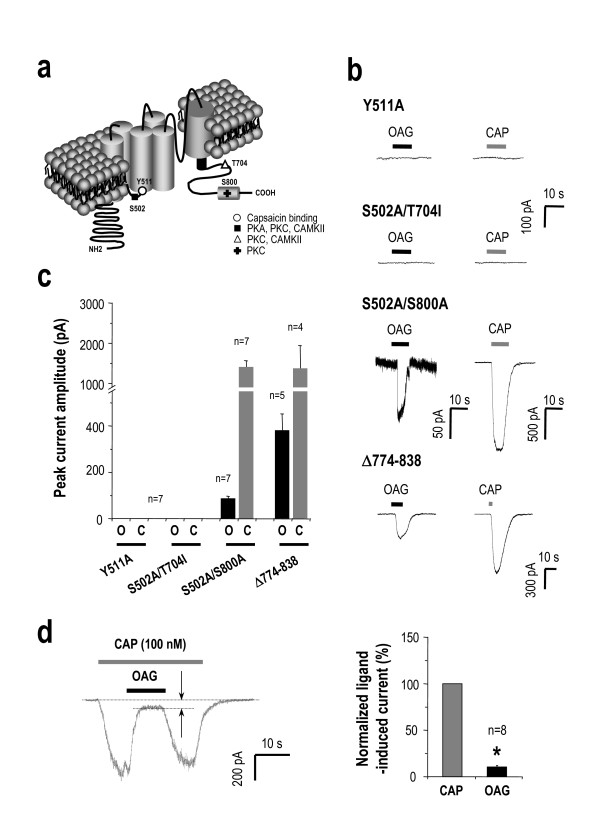
**OAG binds to the capsaicin binding site.** (a) An illustration of the topology of TRPV1. The capsaicin binding site and putative PKC and CaMKII phosphorylation sites were shown. (b) Like capsaicin, OAG failed to activate inward currents in Y511A and S502A/T704I mutants of TRPV1. In contrast, OAG-evoked currents were produced in S502A/S800A and Δ774–838 mutants of TRPV1. (c) Summary of OAG (O) and capsaicin (C) evoked current responses in the TRPV1 mutants indicated in b. (d) Currents evoked by 100 nM capsaicin were partially blocked by 100 μM OAG. The remaining currents between the two arrows were considered to be OAG-induced currents. (e) Summary of OAG (O) and CAP (C) evoked currents. The asterisk showed significant difference in normalized OAG-induced current (paired t-test, p < 0.05).

We next examined the molecular target of DAG on TRPV1 activation. One plausible mechanism of this pathway is DAG directly interacting with TRPV1. To test this possibility we transiently expressed in HEK 293 cells with Y511A mutant of TRPV1 which has impaired capsaicin binding site and found that both OAG and capsaicin failed to elicit a current response (Figure 7b), suggesting that capsaicin and OAG share the binding site at Y511 for activation of TRPV1. Next, we tested whether OAG induce any current in TRPV1 mutants which lack PKC binding sites [28]. Both OAG- and capsaicin-induced currents remained unaffected in double mutations with disrupted PKC binding sites, S502A/S800A (Figure 7b), while the double mutations, S502A/T704I of TRPV1, which lacks the binding site of PKC and Ca^2+^-calmodulin dependent kinase II (CaMKII) were unresponsive to both OAG and capsaicin. These results with mutant analysis suggest that PKC phosphorylation does not play a critical role in the induction of TRPV1 activation by 100 μM OAG, but phosphorylation by CaMKII is required for the OAG-induced activation of TRPV1 [29]. Collectively, our results obtained from TRPV1 mutants suggest that DAG analogs open TRPV1 by binding at Y511 just as capsaicin does and could compete the binding at Y511 site with capsaicin. To directly test this possibility, we applied sub-saturating concentration of capsaicin (100 nM) to induce whole-cell currents and switched to 100 μM OAG in the presence of capsaicin. OAG rapidly and robustly replaced capsaicin, and induced a small inward current (Figure 7d). Taken together, it indicated that OAG and capsaicin share a similar binding site. Our results consistently support the hypothesis that DAG and its analogs activate TRPV1 directly by binding at the capsaicin-binding site. Alternatively, DAG might produce its effects by displacing PtdIns(4,5)P_2 _from an inhibitory site on TRPV1 [30]. Thus, we also tested whether DAG would activate TRPV1 in Δ774–838 deletion mutant of TRPV1, which lacks PtdIns(4,5)P_2 _binding site (786–828) [28]. We observed that OAG induced inward current on HEK 293cell expressing Δ774–838 deletion mutant of TRPV1 (Figure 7b), indicating that OAG-induced activation of TRPV1 is independent of the inhibitory site (774–838) on TRPV1.

## Discussion

Our study proposes that DAG is a novel endogenous ligand of TRPV1 and regulates Ca^2+ ^signaling in rat DRG neurons. It has been well demonstrated that the activation of TRPV1, via GPCRs for bradykinin, histamine, prostaglandin, and serotonin, is associated with sensitization of peripheral nociceptors during thermal and inflammatory hyperalgesia [8,15,16,31,32]. Although lipid metabolite products such as HPETE [7] and anandamide [11] have been suggested as candidate molecules mediating GPCR-activation of TRPV1, an endogenous ligand for TRPV1 in the physiological condition is still debated. In the present study, we found that endogenous DAG produced by GPCR-activation directly activates TRPV1 even in the presence of PKC and DAG lipase inhibitor (Figure 6a). These responses are readily inhibited by capsazepine, a selective TRPV1 blocker (Figure 1b). However, the efficacy of OAG was much less than that of capsaicin, displaying only one fifth of capsaicin induced responses, suggesting that OAG is acting as a partial agonist. Therefore, in addition to TRPC3 TRPC6, and TRPC7, which are activated by DAG [21], we demonstrate that TRPV1 is also activated by DAG and other DAG analogues such as OAG, SAG, and DOG. This result suggested that GPCR-coupled DAG could serve as endogenous ligand of TRPV1 in central nervous system as well as in periphery.

It was reported, using single channel studies of inside-out patches, that TRPV1 is activated by several products of lipoxygenase, but minimally by 1-Stearyl-2-arachinonyl-*sn*-glycerol (SAG), another analog of DAG [7]. This discrepancy might be due to the difference in extracellular Ca^2+ ^levels in the recording conditions and/or the patch clamp recording mode employed. Another report has provided evidence against direct activation of TRPV1 by DAG in a heterologous system utilizing the Chinese Hamster Ovary (CHO) cell line [21]. However, other recent reports demonstrate the complexity of lipid signaling in cell membrane which includes cell-type specificity [33] and different channel activities depending on the mode of patch clamp recording [34].

Our results with whole-cell current recordings in heterologous system indicate that the binding site of DAG might be similar to that of capsaicin. In a competition assay, we found that OAG competitively and rapidly replaced the bound capsaicin, causing reduced whole-cell current amplitude (Figure 7d). These results strongly suggest that DAG binds directly to the capsaicin binding site. In addition, both OAG and capsaicin did not induce any current on the mutant form of TRPV1 with S502A/T704I, which represents the PKC and CaMKII phosphorylation sites. However, OAG and capsaicin induced inward current on the mutant with S502A/S800A, representing the PKC binding sites. These results suggest that like capsaicin, phosphorylation by CaMKII is required for the OAG-activation of TRPV1. Interestingly, in Δ774–838 deletion mutant of TRPV1, which lacks PtdIns(4,5)P_2 _binding site (786–828) (Figure 7b), we observed a bigger inward current by OAG than in wild type TRPV1 (Figure 5Aa) or S502A/S800A mutant (Figure 7b), which is consistent with the previous observation that PtdIns(4,5)P_2 _binding site displays an inhibitory effect of TRPV1 regulation on low concentration of capsaicin [35].

## Conclusion

In summary, the current study demonstrates that DAG is an endogenous ligand for activating TRPV1 by binding at the capsaicin binding site but by acting independently of PKC and lipoxygenase pathway. Our findings should stimulate future investigations in this new pathway of TRPV1 activation, and promise new ways to develop novel drugs for reducing pain.

## Competing interests

The authors declare that they have no competing interests.

## Authors' contributions

DHW performed most of electrophysiology experiments and cobalt staining, and participated in writing this manuscript. SJJ analyzed and interpreted data, assisted data analysis, and designed experiments. MHZ performed the electrophysiology experiments in DRG neurons and HEK 293 cells. CKP performed Ca^2+ ^imaging experiments and RT-PCR. YHK performed Ca^2+ ^imaging and electrophysiology experiments, and participated in data analysis. SBO participated in the design of the study and participated in writing manuscript and CJL wrote this manuscript, designed the experiments, and assisted in data analysis.

## Supplementary Material

Additional file 1 Chelerythrine, a PKC inhibitor, inhibits the tachyphylaxis of capsaicin-induced current. The desensitized current was shown in the subsequent 1 μM capsaicin treatment (left panel) at every 100 s. The inhibition of desensitization was shown in the presence of 1 μM chelerythrine of the internal pipette solution (middle panel). Right panel shows the summary of peak amplitude ratio of second and third divided by the first capsaicin-induced current between in the presence of and in the absence 1 μM chelerythrine. Click here for file
