# Supplementary material for: Direct activation of Transient Receptor Potential Vanilloid 1(TRPV1) by Diacylglycerol (DAG)
Source: Mol Pain. 2008 Oct 1;4:42. doi: 10.1186/1744-8069-4-42 (PMC2576176; doi:10.1186/1744-8069-4-42)
Supplement: Supplementary file 1 [file 1744-8069-4-42-S1.doc]

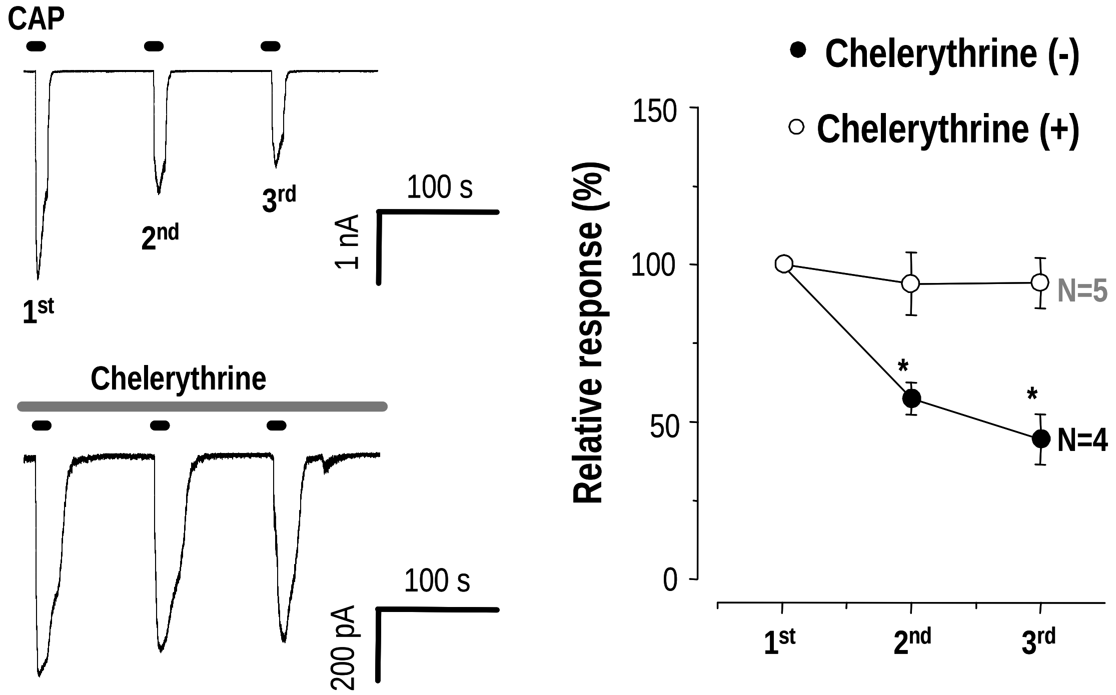

CAP
1st
2nd
3rd
100 s
1 nA
Chelerythrine
100 s
200 pA
Chelerythrine (-)
Chelerythrine (+)
Relative response (%)
150
100
50
0
N=5
N=4
*
*
1st
2nd
3rd
